# Reuse of CD and DVD Wastes as Reinforcement in Gypsum Plaster Plates

**DOI:** 10.3390/ma13040989

**Published:** 2020-02-22

**Authors:** Manuel Alejandro Pedreño-Rojas, María Jesús Morales-Conde, Filomena Pérez-Gálvez, Paloma Rubio-de-Hita

**Affiliations:** Departamento de Construcciones Arquitectónicas 1, Escuela Técnica Superior de Arquitectura, Universidad de Sevilla, Avenida Reina Mercedes, n_ 2, 41012 Sevilla, Spain; mmorales@us.es (M.J.M.-C.); fipergal@us.es (F.P.-G.); palomarubio@us.es (P.R.-d.-H.)

**Keywords:** polycarbonate waste, plastic waste, false ceiling plates, gypsum plasters, mechanical and thermal behaviour

## Abstract

The continuous and rapid evolution in the field of computing, and in particular in the field of storage devices, has led to the obsolescence of optical discs in favour of mass storage devices. In that sense, a large number of CDs and DVDs become obsolete each day in the world. In trying to create a recovery solution for those pieces, research in which polycarbonate (PC) waste from recycled discs have been used to develop new gypsum coating materials and products has been conducted. In a previous study, the physical and mechanical properties of new gypsum plasters, with PC waste as aggregate, were studied. Following that study, this article aims at creating new gypsum plaster false ceiling plates, using CD and DVD residues in different scenarios: as crushed aggregate in the gypsum matrix, as full reinforcement pieces of the plates and as a combination of both. The mechanical behaviour and the thermal conductivity of the new pieces have been analysed in this paper. The results showed an important improvement in the mechanical and thermal properties of the plates when the PC waste was used in many scenarios.

## 1. Introduction

Gypsum plaster is widely used in the construction sector. Mainly, it is used as an interior lining material for walls, partitions and ceilings, and it can be used as a prefabricated plate or as a plaster directly applied onto walls [1]. The gypsum industry has sought to design prefabricated elements with good mechanical behaviour, fundamentally that of strength while being bent, while also having adequate thermal and acoustic properties [2,3,4].

On the other hand, the continuous and rapid evolution in the field of computing, and in particular in the field of storage devices, has led to the obsolescence of optical discs (CDs and DVDs) in favour of mass storage devices. In addition, in Spain, approximately 100,000 CDs become obsolete each month and end up in landfills or incinerators because the data they contain simply ceases to be useful [5]. They are made of synthetic materials, of which around 95% is plastic, in the form of polycarbonate (PC), which is essential for its high optical quality [6].

Trying to achieve an improvement in the bending strength of gypsum products, several researchers have analysed the possibility of incorporating various types of fibres or aggregates to the gypsum matrix. Of all of them, the ones that used glass fiber in the development of gypsum plates stand out, with this fibre being a fundamental component of the majority of plasterboards now available on the market. Ali and Grimer were some of the first to study the influence of E-glass fibres in gypsum composites. In their study, they realised that by making the reinforcement, a better flexural, tensile, compression and impact strength was achieved [7]. Other researchers used the glass fibre reinforcement to develop a water-resistant gypsum panel [8]. Del Río-Merino et al. studied the influence of a glass fibre reinforcement in the physical and mechanical properties of gypsum plasters [9]. Recently, Rodriguez-Liñán et al. compared the effect of gypsum plasters reinforcement with glass fibre and straw, obtaining better results when the former was used [10].

Furthermore, many studies have been conducted recently in which different kinds of plastic waste were added as aggregates to a gypsum matrix to try to improve some of their properties. One of the most used was polyethylene terephthalate (PET), which is present in numerous objects used in daily life (bottles, brushes, strings, etc.). Some authors confirmed that by increasing the percentage of PET added to the plaster (up to 20%), lighter composites with improved thermal properties were obtained, but to the detriment of their mechanical behaviour [11]. Contributions that used foamed plastic waste as aggregates to the gypsum matrix to develop the new plasters, are noteworthy [12]. San Antonio-González et al. analysed the effects of using extruded and expanded polystyrene (XPS and EPS) waste to create new gypsum composites. They concluded that the lightness and thermal properties of the plaster were improved, and the composite also achieved the minimum standard requirements [13] for those materials [14,15]. Polyurethane foam (PUR) waste was also used as aggregate in gypsum plasters. In this case, the authors tried to achieve the maximum volume ratio of PUR/plaster without losing their mechanical requirements. They obtained lighter composites with improved thermal conductivity, while other properties such as adherence and mechanical strength decreased [16].

There is other research that has led to the development of new lightweight construction materials as a result of adding different plastic aggregates, for example: PVC pipes [17], plastic bag waste [18], rubber particles from recycled tyres [19], plastic from the recycling of electronic devices [20], polyethylene waste [21] and diatomite and polypropylene fibres [22].

Finally, some new gypsum products (plates, blocks, panels, etc.) have been developed using several kinds of waste [23,24,25]. The contribution made by Pedreño-Rojas et al. is noteworthy, as they created new wood waste-gypsum false ceiling plates with improved thermal and acoustic properties [26].

There are very few previous localized accounts of using CD waste as aggregate in the development of new construction materials. Only two contributions that employed the waste as a fine aggregate replacement in the development of new concretes are remarkable [27,28].

This paper is the second part of research in which PC wastes from recycled CDs and DVDs have been used to develop new gypsum coating materials and products. Previously, the physical and mechanical properties of new gypsum plasters, with PC waste as aggregate, were studied [29]. Continuing from that research, this article aims at creating new gypsum plaster false ceiling plates, using the CD and DVD residue in different ways: as crushed aggregate in the gypsum matrix, as full reinforcement pieces of the plates, and as a combination of both. The mechanical behaviour and the thermal conductivity of the new pieces have been analyzed in this paper.

## 2. Materials and Methods 

### 2.1. Materials

#### 2.1.1. Gypsum

For the development of the new false ceiling gypsum plates, traditional commercial gypsum for construction (B1) was used [13]. Its main properties are presented in Table 1.

#### 2.1.2. Polycarbonate (PC) Waste from Recycled CDs and DVDs

All the CDs and DVDs used this research were collected from different recycling points in the University of Seville, Spain. Table 2 shows the mechanical properties of a compact disc, according to the data obtained by Ibrahim et al. [30]. Other studies confirmed that the strength capacity of recycled PC from CDs and DVDs is only 20% lower than that of virgin polycarbonate [31]. 

**Figure 1:**
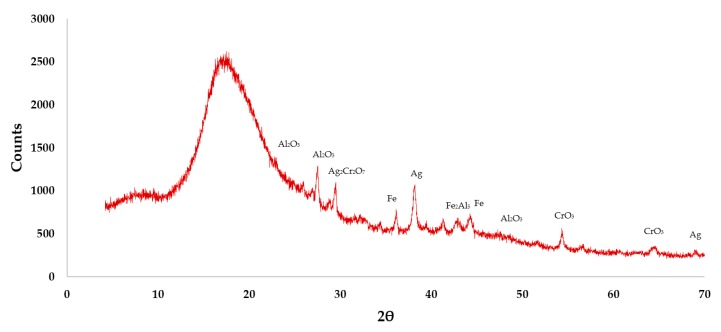
XRD analysis of the polycarbonate waste used.

The chemical composition of the recycled discs was obtained using the X-ray diffraction (XRD) technique (Figure 1). It was found that the sample was 69.8% amorphous, with the remaining 30.2% being crystalline (Table 3). 

**Figure 2:**
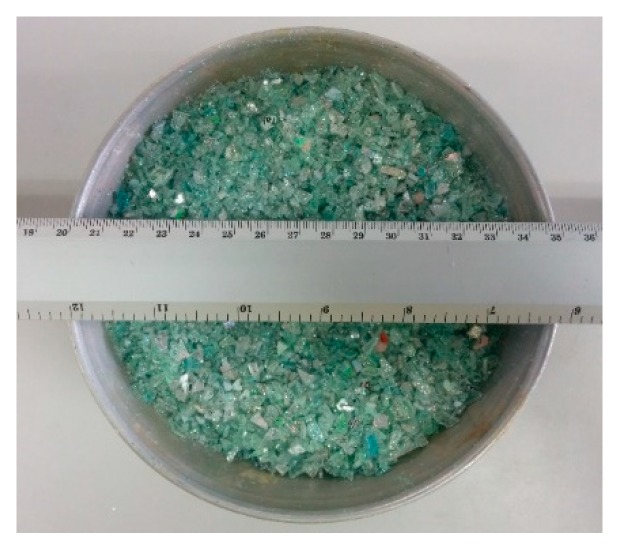
Shredded CDs and DVDs ready to be used as aggregate in the mixtures.

**Figure 3:**
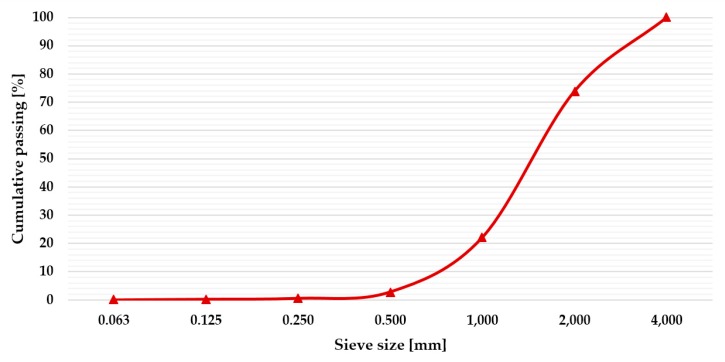
Particle size distribution of crushed discs used in the mixtures.

The CDs and DVDs were used in two different ways to develop the new plates: as full pieces, and as a fine aggregate for the gypsum matrix. To crush the discs, an Enviro EN RS machine was used, obtaining pieces smaller than 4 mm, as can be seen in Figure 2. The granulometry of the crushed discs is presented in Figure 3.

### 2.2. Samples Definition

In accordance with the regulations, the false ceilings plates had a side measurement of 600 mm with a 15 mm thickness [32]. Eleven series of plates and three plates per series were prepared, giving 33 test samples, according to different scenarios.

#### 2.2.1. SCENARIO 1: Crushed PC Waste Used as Aggregate in the Gypsum Matrix

In accordance with the results obtained in the first stage of this research [29], four different series of gypsum plates with crushed discs aggregate were prepared according to the compositions described in Table 4. It must be noted that the Reference sample (the one without any type of waste) is the same for all the described scenarios.

#### 2.2.2. SCENARIO 2: Full CDs Used as Internal Reinforcement in the Gypsum Plates

**Figure 4:**
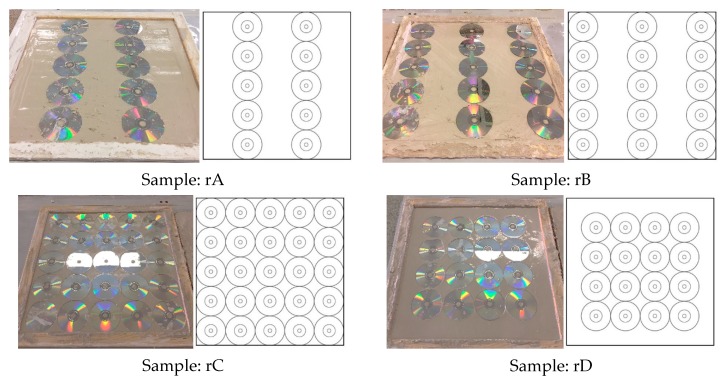
Test plates prepared for Scenario 2.

For the second scenario, an internal layer of full CDs (with four different dispositions) was placed in the centre of the plates. Apart from the discs’ reinforcement, the plates have the same composition as the Reference sample described in Table 4 (7000 g of gypsum and 3850 mL of water). The various CD reinforcement dispositions are defined in Figure 4.

#### 2.2.3. Scenario 3: Full CDs Internal Reinforcement and Crushed PC Waste Aggregate in the Matrix 

After observing the mechanical behavior of the plates developed for Scenarios 1 and 2, a new scenario combining the previous ones was created. Two new series were developed: the first with the same composition of the PC20 plate and the second with the same composition as the PC40 plate. For both, as the internal reinforcement for the CDs, the disposition rD was used. 

### 2.3. Test methods

#### 2.3.1. PC Waste-Gypsum Plasters Previous Physical and Mechanical Characterization

As mentioned, previously, the new gypsum plasters with PC waste aggregate were characterized [29]. Their density, flexural strength, compressive strength and elastic modulus (E) were obtained by following the procedure described in UNE-EN 13279-2 [33].

#### 2.3.2. Flexural Strength Test

**Figure 5:**
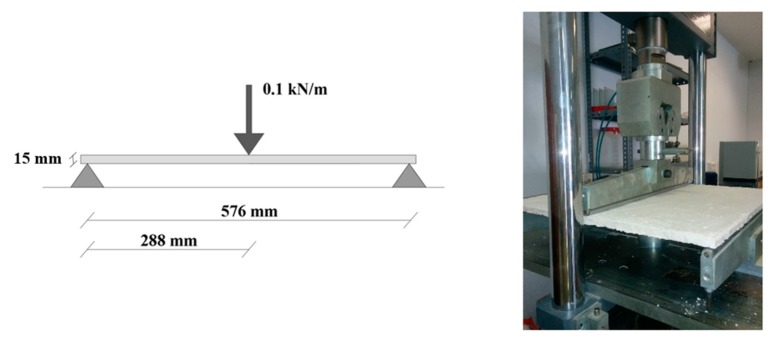
Flexural strength test.

After preparation, the plates were preserved for seven days in a curing chamber at 23 ± 2 °C and at a relative humidity of 50 ± 5%. After seven days, the specimens were placed in an oven for 24 h at 40 ± 2 °C to constant weight [32] and tested for flexural strength. In this test, each plate had to bear a uniform linear load of 6 kg (0.1 kN/m) on its central fiber (Figure 5). The plates must withstand this stress for 30 min without visible signs of damage [32]. After this test, the plates were tested to breaking point to obtain the breaking load value for each one.

#### 2.3.3. Thermal Conductivity Test

Before they were broken, the thermal conductivity of each plate was obtained using the ISOMET-2114 device operating in transient mode, following the procedure described in ASTM D5930 [34], which involved testing for 45 min for each measurement at a dry state.

## 3. Results and Discussion

### 3.1. Previous Tests Results

The density and the mechanical behavior (flexural and compressive strength and elastic modulus E) of the new PC waste-gypsum plasters were measured [29]. Table 5 presents the numerical results obtained from the tests performed with their coefficient of variation (CoV (%)).

As can be observed, the composites were lighter when the percentage of plastic waste added to them increased. Furthermore, an important improvement on the compressive strength results was obtained for most of the mixtures (up to 34.5%). Finally, the plaster PC10 presented a slightly higher flexural strength value than the reference material.

### 3.2. Weight of the Various Plates under Study

**Figure 6:**
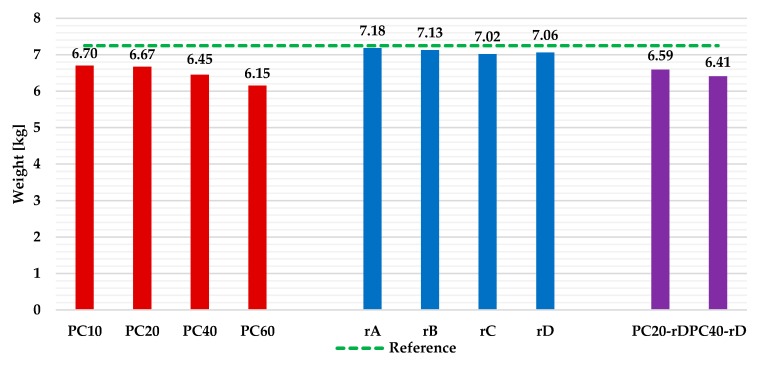
Weight measurement of the plates.

All the plates, before being submitted to the flexural strength test, were weighed on a precision scale. The weight results are presented in Figure 6.

As can be noticed, in all of the scenarios, each plate was lighter than the reference plate. That reduction was higher when the percentage of crushed PC added to the matrix increased (up to 15.2%), with the reduction being insignificant for the plates in Scenario 2 (3.2%).

### 3.3. Flexural Strength Test Results

The flexural strength test results for the reference plates are shown in Table 6. As can be noticed, all of the plates satisfactorily passed the standard requirement.

#### 3.3.1. Scenario 1

The flexural strength test results for the plates in Scenario 1 are presented in Table 7.

**Figure 7:**
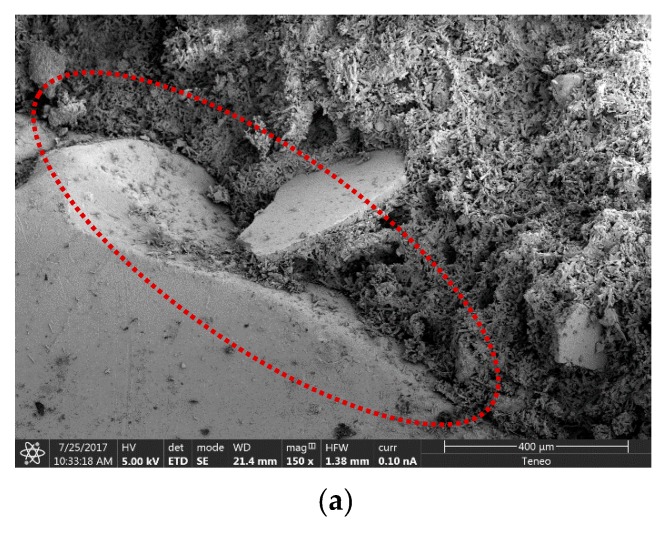

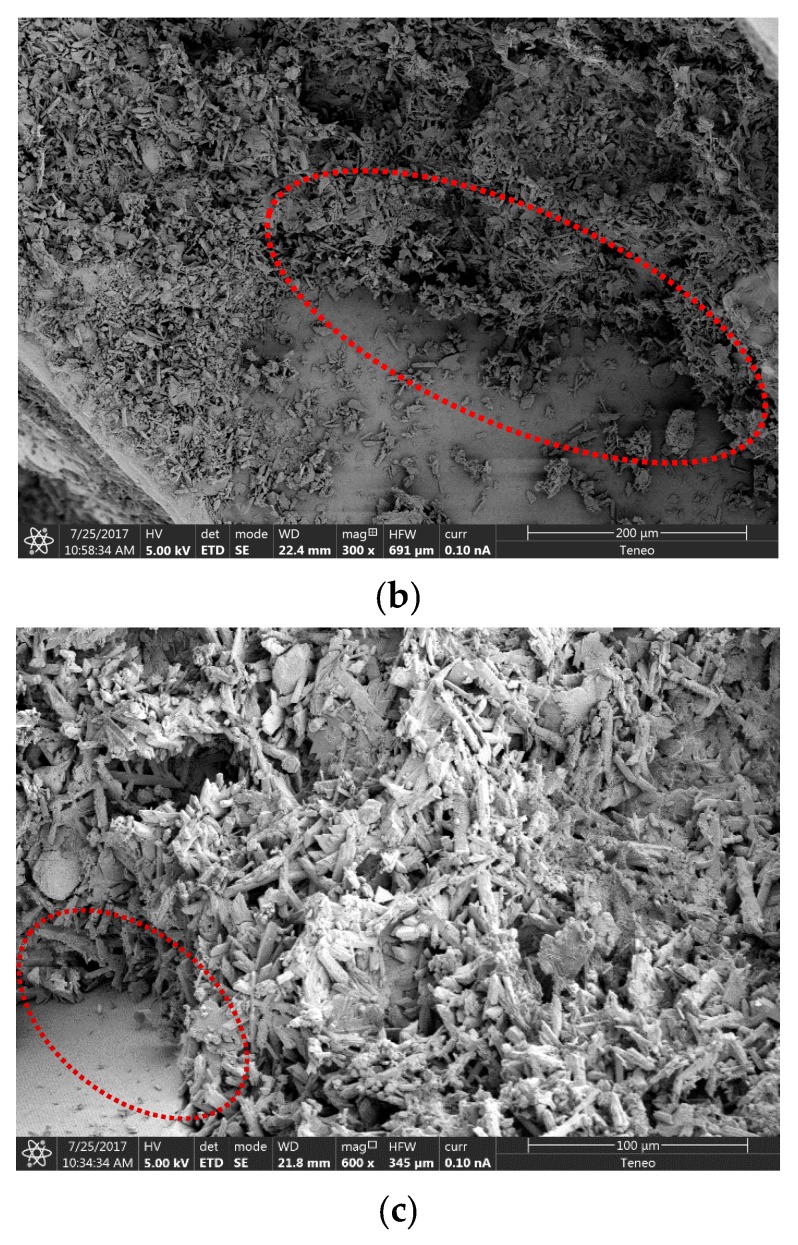
SEM images of the PC40 sample, showing a good adherence between the PC waste and the gypsum matrix: (**a**) 150×; (**b**) 300×; (**c**) 600×.

According to the results achieved, a slight improvement on the bending behaviour of the PC10 plates was obtained (8.7% higher). Those results are in accordance with the ones obtained during the mechanical characterization of the new plasters [29]. In general terms, excellent mechanical behavior was achieved for all the plates, even in those that contain 60% of PC waste added to their mixture. The good performance of the new plates can be justified by the good adherence obtained between the plastic waste and the gypsum matrix, as can be seen in the scanning electron microscop (SEM) images of Figure 7.

#### 3.3.2. Scenario 2

The flexural strength test results for the plates in Scenario 2 are presented in Table 8.

According to the results achieved, the rD disposition was the only one that significantly improved the mechanical performance of the reference plates (up to 19.3%). On the other hand, for all of the other dispositions, a worsening resulted, with all of them passing 6 kg during a 30 min standard requirement. The lowest values were achieved by the rC plates, decreasing the flexural strength by up to 52.6% compared to the reference samples. The arrangement of CDs next to the edges of the plate caused both layers of the plaster matrix not to work in solidarity, since there was not enough contact surface between them, thus causing a significant drop in the resistance values.

#### 3.3.3. Scenario 3

After finding that the rD disposition was the one that achieved the highest flexural strength, Scenario 3 plates were developed using that reinforcement and with the PC20 and PC40 matrix composition. The flexural strength test results for the plates in Scenario 3 are presented in Table 9.

The test results showed an important improvement on the flexural strength of the PC20-rD plates compared to the reference sample (up to 26.3%). Moreover, a good mechanical performance was achieved for the PC40-rD samples. 

#### 3.3.4. Final Discussion

**Figure 8:**
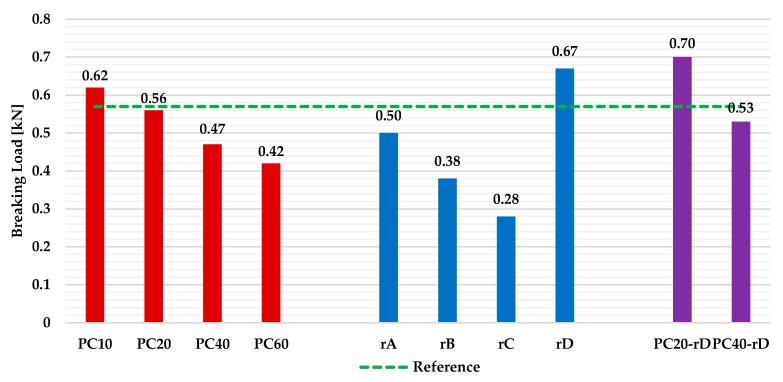
Breaking load results for all the scenarios (plates) under study.

A comparison of the flexural strength test results for all the scenarios is presented in Figure 8.

The values showed that, in general terms, the best performance was achieved by the Scenario 3 plates, with the PC20-rD samples the ones that obtained the highest value (0.70 kN). It is important to note that the three series increased the flexural strength value of the reference specimens (PC10, rD and PC20-rD plates). On the other hand, the lowest result was achieved by the rC samples. Comparing the results of the Scenarios 1 and 2 with the ones obtained for the Scenario 3, it can be appreciated that a better mechanical performance was achieved when both reinforcement options worked together. In the case of the PC20 plates, the increase when adding the reinforcement with full CD pieces was 25%, while the increase for the PC40 series was 12.8%. To sum up, it should be considered that, despite not having the best resistance results in some series, the plates that incorporated the residue into the gypsum matrix (Scenarios 1 and 3) have a better environmental performance. This is since, in addition to reusing large amounts of waste, they substantially reduce the amount of commercial gypsum used to make each plate [26].

**Figure 9:**
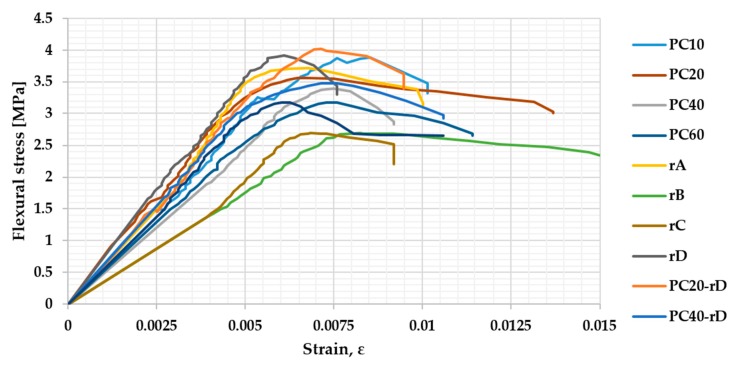
Stress–strain plots.

The stress–strain plots are presented below to show the effect of the reinforcement and the work of the fracture (Figure 9).

### 3.4. Thermal Conductivity Test Results

**Figure 10:**
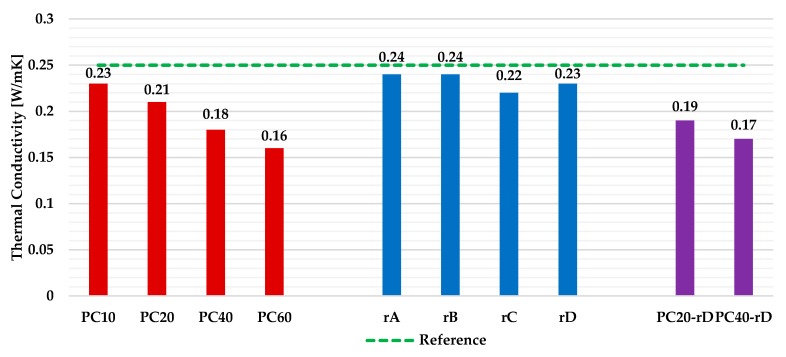
Thermal conductivity of the various gypsum plates under study.

The thermal conductivity test results for all the plates are shown in Figure 10. 

The provided results show that, when the amount of PC waste added to the plates increased, the thermal conductivity of the composites decreased. The specimens that contained plastic aggregate in the matrix (scenarios 1 and 3) were the ones that achieved the best thermal performance. The best thermal conductivity value (0.16 W/mK) was achieved by the PC60 plates, in which a reduction of 36% was obtained compared to the reference samples. On the other hand, despite slightly decreasing the conductivity values, the worst results were achieved by the Scenario 2 samples, since the amount of plastic residue contained in them was minimal compared to the other series. The achieved results are in accordance with other previous research, which demonstrated that the amount of plastic waste added to the gypsum matrix improved the thermal behavior of the plaster [14,16].

## 4. Conclusions

In this research, the influence of recycled CDs and DVDs as a reinforcement in gypsum plaster false ceiling plates was analysed. Three different reinforcement scenarios were studied: crushed discs used as aggregates in the gypsum matrixes, full CDs as internal reinforcement of the plates, and a combination of both. The flexural strength and the thermal conductivity of the new pieces were measured. According to the achieved results, the following conclusions were drawn:

- For all the scenarios, the use of the recycled discs was linked to a decrease in their weight. The lightest plates were the PC60 series samples (15.2% lower than the reference samples).
- According to the flexural strength test results, in general terms, the best performance was achieved by the Scenario 3 plates, with the PC20-rD samples the ones that obtained the highest value (0.70 kN). On the other hand, the lowest result was achieved by the rC samples. Comparing the results of the Scenarios 1 and 2 with the ones obtained for the Scenario 3, it can be appreciated that a better mechanical performance was achieved when both reinforcement options worked together.
- The thermal test results showed that, when the amount of PC waste added to the plates increased, the thermal conductivity of the composites decreased. The specimens that contained plastic aggregate in the matrix (Scenarios 1 and 3) were the ones that presented the best thermal performance. The best thermal conductivity value (0.16 W/mK) was achieved for the PC60 plates.

To sum up, it can be said that the reuse of PC waste from recycled optical discs in gypsum plaster plates offered several advantages compared to the reference product. All the newly developed plates are lighter and possess improved thermal behaviour, and some of them also increased the flexural strength capacity of the pieces.

## Figures and Tables

**Table 1:** Properties of B1 gypsum used as base material.

Purity [%]	Granulometry [mm]	Surface Hardness [Shore C]	Performance [kg/m^2^/cm]	Flexural Strength [N/mm^2^]	Compressive Strength [N/mm^2^]	Adherence [N/mm^2^]	Ph
>75	0–1	≥45	10–12	≥2	≥2	>0.1	>6

**Table 2:** Mechanical properties of a compact disc [28].

Sample	Thickness [mm]	Tensile Strength [MPa]	Tensile Modulus [MPa]	Elongation at Break [%]	Compressive Strength [MPa]	Compressive Modulus [MPa]
**CD0**	1.42	32.7	972.5	120	82.7	1654.7

**Table 3:** Crystalline phases of the disc waste used.

Element	Al_2_O_3_	Fe_2_Al_5_	Fe	FeO	Ag	CrO_3_	Ag_2_Cr_2_O_7_
(%)	29.08	9.38	15.85	11.38	16.77	6.92	10.62

**Table 4:** Test plates prepared for the Scenario 1.

Samples	Gypsum [g]	Water [g] [mL]	Crushed PC Waste [g]	Reinforcement Volume [% by Volume of Gypsum]
Reference	7000	3850	-	-
PC10	6000	3300	600	17.73
PC20	5000	2750	1000	35.47
PC40	4000	2200	1600	70.93
PC60	3500	1925	2100	106.4

**Table 5:** Previous test results [29].

Sample Series	Density [g/cm^3^] (CoV [%])	Flexural Strength [MPa] (CoV [%])	Compressive Strength [MPa] (CoV [%])	E [MPa] (CoV [%])
Reference	1.336 (0.69)	3.420 (7.36)	6.680 (5.26)	697.46 (4.43)
PC10	1.242 (1.14)	3.541 (10.90)	8.984 (5.27)	704.17 (5.80)
PC20	1.235 (1.56)	3.054 (13.29)	8.036 (10.01)	681.25 (9.35)
PC40	1.194 (1.73)	2.617 (8.89)	7.701 (7.23)	515.38 (7.49)
PC60	1.138 (2.01)	2.328 (12.33)	5.590 (9.39)	316.67 (9.11)

**Table 6:** Flexural strength test results for the reference plates.

Plate	UNE-EN 14246 Check (6 kg 30’)	Breaking Load [kN]	Mean Value [kN]
Reference 1	OK	0.61	0.57
Reference 2	OK	0.57	
Reference 3	OK	0.54	

**Table 7:** Flexural strength test results for the Scenario 1 plates.

Plate	UNE-EN 14246 Check (6 kg 30´)	Breaking Load [kN]	Mean Value [kN]
PC10 1	OK	0.64	0.62
PC10 2	OK	0.63	
PC10 3	OK	0.59	
PC20 1	OK	0.58	0.56
PC20 2	OK	0.55	
PC20 3	OK	0.54	
PC40 1	OK	0.49	0.47
PC40 2	OK	0.46	
PC40 3	OK	0.45	
PC60 1	OK	0.44	0.42
PC60 2	OK	0.41	
PC60 3	OK	0.41	

**Table 8:** Flexural strength test results for the Scenario 2 plates.

Plate	UNE-EN 14246 Check (6kg 30´)	Breaking Load [kN]	Mean Value [kN]
rA 1	OK	0.53	0.50
rA 2	OK	0.49	
rA 3	OK	0.48	
rB 1	OK	0.41	0.38
rB 2	OK	0.37	
rB 3	OK	0.35	
rC 1	OK	0.30	0.28
rC 2	OK	0.28	
rC 3	OK	0.27	
rD 1	OK	0.68	0.67
rD 2	OK	0.68	
rD 3	OK	0.66	

**Table 9:** Flexural strength test results for the Scenario 3 plates.

Plate	UNE-EN 14246 Check (6 kg 30’)	Breaking Load [kN]	Mean Value [kN]
PC20-rD 1	OK	0.72	0.70
PC20-rD 2	OK	0.70	
PC20-rD 3	OK	0.67	
PC40-rD 1	OK	0.56	0.53
PC40-rD 2	OK	0.52	
PC40-rD 3	OK	0.51	
